# The effect of high-throughput vitrification of human ovarian cortex tissue on follicular viability: a promising alternative to conventional slow freezing?

**DOI:** 10.1007/s00404-022-06797-6

**Published:** 2022-09-29

**Authors:** Andreas Schallmoser, Rebekka Einenkel, Cara Färber, Norah Emrich, Julia John, Nicole Sänger

**Affiliations:** Department of Gynecological Endocrinology and Reproductive Medicine, University Hospital of Bonn, Venusberg Campus 1, 53127 Bonn, Germany

**Keywords:** Ovarian tissue, Slow freezing, Vitrification, Fertility protection, Ovary, Follicle

## Abstract

**Background:**

The standard procedure most frequently used for ovarian tissue cryopreservation (OTC) is slow freezing, while vitrification has been proposed as promising alternative and has built an impressive catalog of success in fertility laboratories regarding cryopreservation of oocytes and embryos.

**Methods:**

We developed and evaluated a high-throughput protocol for vitrification of human ovarian tissue suitable for clinical processing. Follicular viability was assessed via calcein staining prior and after cryopreservation analyzing ovarian tissue of a cohort of 30 patients.

**Results:**

We found no significant differences regarding follicular viability between slow frozen and vitrified cortex tissue samples 24 h after thawing and rapid warming. Follicular viability of thawed and rapid warmed samples was not significantly different in comparison to fresh samples, indicating high proportions of follicular survival rates with both methods.

**Conclusions:**

High-throughput vitrification is a promising option in a clinical setting. More research is required to determine the status of other tissue-specific quality indicators potentially influencing on autotransplantation.

## What does this study add to the clinical work

**Table:** 

In summary, our results indicate that rapid vertical vitrification of ovarian tissue may be equivalent to slow freezing in terms of follicular viability while offering a cost efficient alternative to conventional slow freezing procedures.	

## Introduction

Cryopreservation of human oocytes and embryos is a well-established routine method. In many IVF clinics, slow freezing has been superseded by vitrification due to superior survival rates [1–4] accompanied by considerable technical simplification. Additionally, with slow frozen cells, it was shown that applying rapid warming gives superior results compared to standard thawing protocols in terms of survival rates [5–7]. Preservation of oocytes and ovarian tissue cryopreservation (OTC) are two options for fertility protection in female cancer patients [8–10]. Multiple working groups have reported pregnancies and live births after thawing and transplantation of cryopreserved ovarian tissue, substantiating that OTC is a promising alternate method of fertility protection [11–19]. The large majority of OTC data are based on the slow freezing method, that is regarded as well established [20]. Vitrification of ovarian tissue is a promising alternative method with potential major advantages [21–25]. However, the number of reports about pregnancies and live births after retransplantation of vitrified ovarian tissue is still limited [20, 26]. The majority of centers performing OTC use the slow freezing method while the optimal protocol regarding vitrification of ovarian tissue has yet to be determined.

## Methods

### Ethics

Ethics committee of University Hospital Bonn approved this study (007/09) including the use of 10% of the ovarian tissue for patient related research and quality control analysis. Written, informed consent was obtained individually from each patient.

### Statistics

**Fig. 1:**
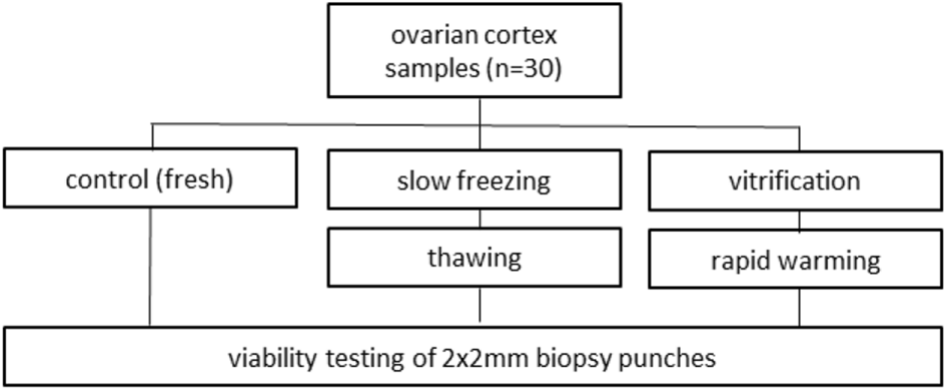
Experimental design of follicular viability measurements

Data were analyzed with SPSS version 25 (IBM) statistical software. Wilcoxon test was used to compare parameters between related samples. Mann–Whitney *U* test was conducted to determine parameters between groups. Data were tested for normal distribution with Kolmogorov–Smirnov test (Fig. 1).

### Ovarian tissue

Ovarian tissue was obtained from 30 patients aged 14–41 (Ø 26.7) years prior cryopreservation for fertility protection measures.

### Preparation of material for vitrification

**Fig. 2:**
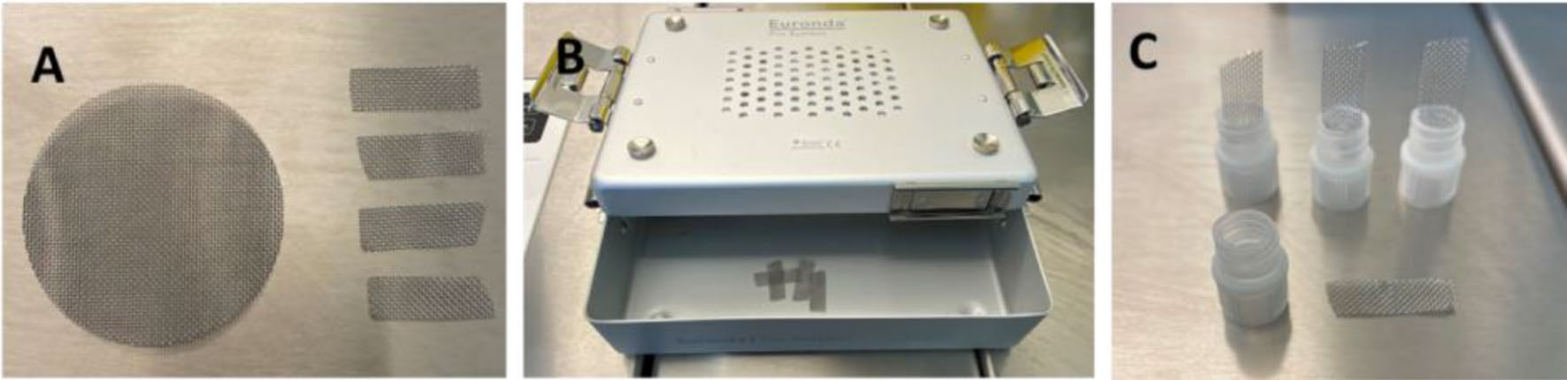
Preparation of material. Metal cell meshes were cut to 25 × 8 mm stripes (**A**) and prepared for autoclaving (**B**). After sterilization, meshes were linked with caps of 1.8 ml vials (**C**) under sterile air conditions

Metal cell meshes (Merck, Darmstadt) with opening sizes of 380 µm were customized to 25 × 8 mm stripes (Fig. 2A). Devices were placed in a sterilization container (Fig. 2B, Euronda Eurobox) prior autoclaving (Tuttnauer, Breda, Netherlands) under humid heat [27, 28] to sterilize the meshes prior further processing. Customized stripes were linked under validated sterile laminar air flow conditions (Kendro Hera Safe) with the caps of 1.8 ml cryovials (Thermo Scientific, Waltham, USA) to obtain thermally conductive loading devices (Fig. 2C).

### Surgical retrieval and tissue preparation for cryopreservation

**Fig. 3:**
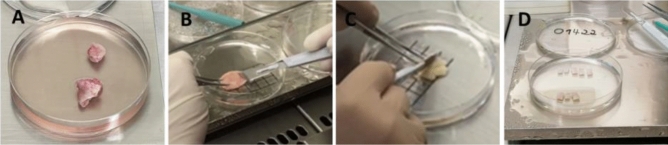
Preparation of ovarian tissue. Ovarian tissue (**A**) was processed by removal of the medulla (**B**) and customized (**C**) to 10 × 5 × 1 mm stripes (**D**) for cryopreservation

Ovarian tissue was surgically removed prior initiation of gonadotoxic cancer treatment or due to a disease that causes premature limitation of the ovarian reserve. Excision of ovarian tissue was performed laparoscopically in an outpatient surgery. Ovarian tissue resection was performed with sharp scissors without any electrocoagulation to protect oocytes. 30–50% (Fig. 3A) of one ovary was resected. Directly after removal, the tissue was transferred to a tube with custodiol solution (Dr. Köhler, Bensheim, Germany) at 4 °C and forwarded to the lab without deviation. A small part of the tissue was conserved in formalin solution and sent to histopathology to investigate for abnormalities, especially for cancer metastases. After ablation of the medulla (Fig. 3B), cortex was processed in stripes measuring 10 × 5 × 1 mm (Fig. 3C, D) under validated sterile laminar air flow conditions to ensure the highest level of safety and quality accompanying the manufacturing process. Cortex preparation was performed in custodiol (Dr. Köhler, Bensheim, Germany) at 4 °C. Additionally, 2 × 2 mm biopsy punches (pfm medical, Köln, Germany) were prepared for standardized viability measurement pre cryopreservation and post thawing/rapid warming. Cryopreservation was defined as exposition of prepared tissue to cryoprotective agents (CPA) and completion of cooling procedure before storing the samples in the cryobank.

### Decontamination of liquid nitrogen

Liquid nitrogen was sterilized according to established protocols [29–31]. In brief, nitrogen was exposed to UV irradiation at 254 nm (OSRAM Germicidial Puritec, Berlin, Germany) at a distance of 20 cm for 20 min under a validated laminar flow bench.

### Vitrification

**Fig. 4:**
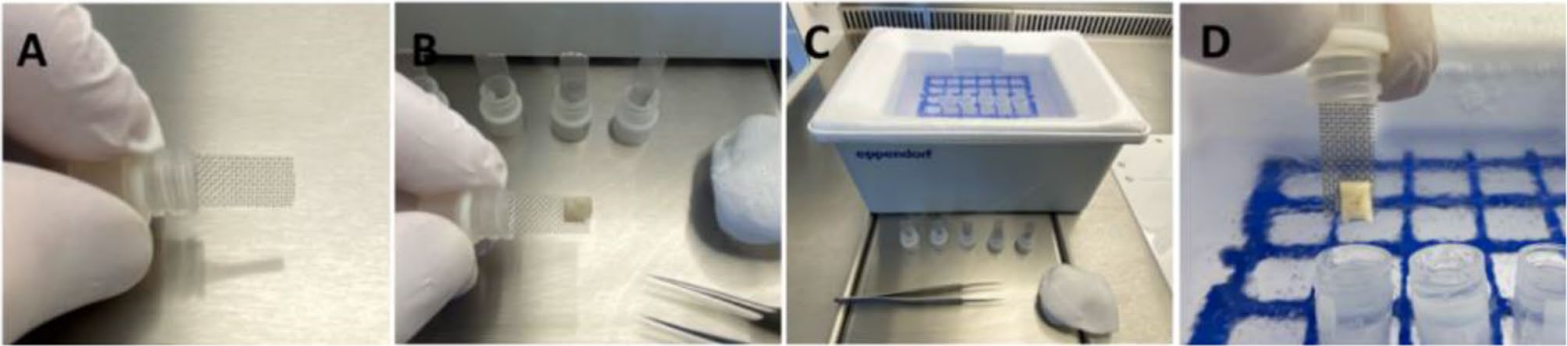
High-throughput vitrification of ovarian tissue. Ovarian cortex tissue placed on a loading device (**A**, **B**) for rapid vertical vitrification (**D**) in a vial based closed system on a customized, grid based styrofoam device (**C**), capable of high sample throughput

Vitrification and rapid warming media was prepared according to Suzuki and colleagues with modifications [26]. In brief, tissue was equilibrated in GMOPS + (Vitrolife, Göteborg, Sweden) supplemented with 10% serum substitute supplement [SSS] (Fujifilm Irvine scientific, Santa Ana, USA) and 10% ethyleneglycol (Merck, Darmstadt, Germany) for 5 min (step 1), followed by equilibration in GMOPS + (Vitrolife, Sweden) supplemented with 10% SSS (Fujifilm Irvine scientific, Santa Ana, USA) and 20% ethyleneglycol (Merck, Darmstadt, Germany) for 5 min (step 2). Finally, samples were transferred to GMOPS + (Vitrolife, Göteborg, Sweden) supplemented with 10% SSS (Fujifilm Irvine scientific, Santa Ana, USA), 35% ethyleneglycol (Merck, Darmstadt, Germany), 5% polyvinylpyrrolidone [PVP] (Merck, Darmstadt, Germany) and 0.5 mol/L sucrose (Merck, Darmstadt, Germany) [step 3]. After 6 min and within 7 min, tissue pieces were placed on the loading device (Fig. 4A, B), surplus liquid was dabbed away with sterile cellulose material. Samples were vertically plunged in cryovials pre-filled with liquid nitrogen (Fig. 4D) in a setting prepared for high throughput, arranged on a loading grid within a customized cryo dewar vessel (Fig. 4C, Eppendorf, Hamburg, Germany). Equilibration steps 1–3 were performed in a 6-well dish (Sarstedt, Germany) with 5 ml media/well on a rocking shaker at room temperature under validated sterile laminar air flow conditions (Kendro Hera Safe, Heraeus).

### Rapid warming

**Fig. 5:**
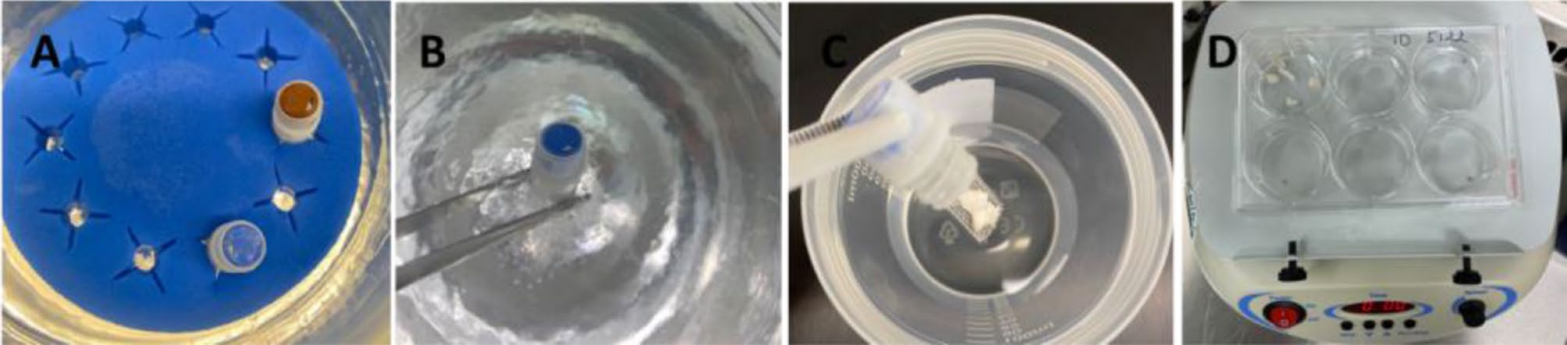
Rapid warming. Cryovials arranged on a floater (**A**), ready for warming. Cryovials were opened cautiously (**B**) and grids were submerged (**C**) rapidly in 30 ml of warming solution 1, transferred to warming solution 2 and washing solutions 1 and 2 on a rocking shaker (**D**)

Cryovials were placed on a floater (Fig. 5A) in a dewar vessel filled with liquid nitrogen. Vials were opened cautiously (Fig. 5B) and metal meshes with cortex strips were submerged (Fig. 5C) rapidly in 30 ml of prewarmed (37.2 °C) GMOPS + (Vitrolife, Göteborg, Sweden) media supplemented with 0.8 mol/l sucrose (Merck, Darmstadt, Germany) and 10% SSS (Fujifilm Irvine scientific, Santa Ana, USA) for 1 min (step 1) followed by incubation in GMOPS + (Vitrolife, Göteborg, Sweden) media supplemented with 0.4 mol/l sucrose (Merck, Darmstadt, Germany) and 10% SSS (Fujifilm Irvine scientific, Santa Ana, USA) for 3 min (step 2). Finally, 2 washing steps (steps 3–4) with GMOPS + (Vitrolife, Göteborg, Sweden) supplemented with 10% SSS (Fujifilm Irvine scientific, Santa Ana, USA) for 5 min each were conducted. Warming steps 1–4 were performed under validated sterile laminar air flow conditions. Step 1 was performed in a prewarmed sterile cup (Sarstedt, Nümbrecht) at 37.2 °C on a heating plate, while steps 2–4 were conducted in a 6-well dish (Sarstedt, Nümbrecht, Germany) with 5 ml media/well on a rocking shaker (Fig. 5D) at room temperature.

### Slow freezing and thawing

Cryopreservation and thawing of cortex samples were performed according to established protocols with modifications [32–37]. In brief, tissue samples were incubated in L-15 Leibovitz’s medium (Gibco Life technologies, NY, U.S.A.) supplemented with 11% human serum albumin [HSA] (Irvine Scientific, Santa Ana, USA), 10% dimethyl sulfoxide [DMSO] (CryoSure DMSO, WAK Chemie, Steinbach, Germany) and equilibrated for 35 min prior slow freezing procedure. Cortex samples were transferred to 1.8 ml vials (Nunc, Thermo Fisher Scientific, Denmark) and cryopreserved with a cooling rate of − 2 °C per min. After seeding, samples were cooled at a rate of − 0.3 °C per min to − 40 °C and at − 10 °C per min to − 140 °C. For thawing, tissue samples were brought to room temperature for 40 s and kept in a water bath for 130 s at 37.2 °C. Transfer of tissue was conducted in 15-min steps to 3 thawing solutions of decreasing sucrose concentrations based on Dulbecco’s phosphate buffered saline [DPBS] (DPBS CTS, Gibco Life technologies, NY, U.S.A.) supplemented with 11% HSA (Irvine Scientific, Santa Ana, USA) prior 2 washing intervals.

### Determination of follicular viability

**Fig. 6:**
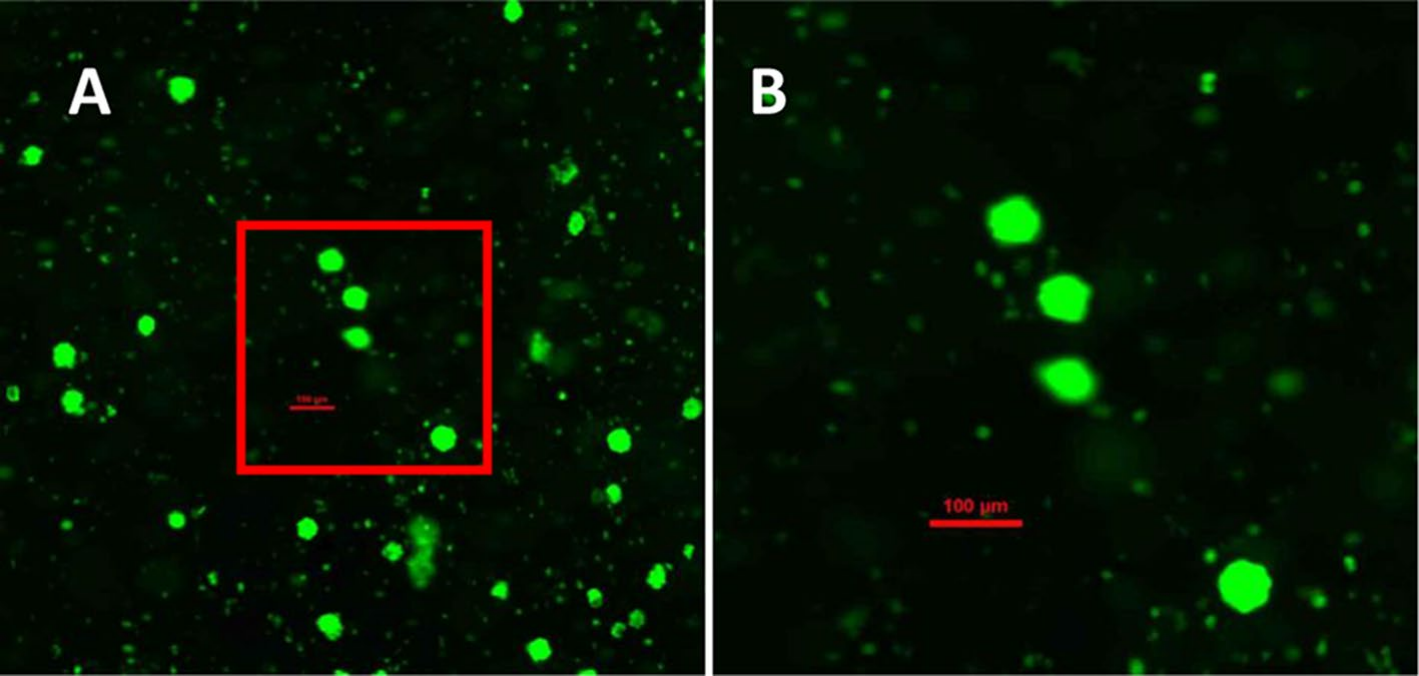
Viability staining of ovarian cortex samples after digestion with collagenase. Viable follicles can be identified by a layer of granulosa cells enclosing the oocyte emitting green fluorescence (495 nm) and by their size difference to stroma cells (**A**, **B**). Picture with tenfold (**A**) magnification displaying viable follicles. Zoomed in section of A with 4 viable follicles (**B**) at 20-fold magnification. Scalebar 100 µm

Calcein AM (Merck, Darmstadt, Germany) was dissolved with DMSO (Cryosure DMSO, WAK Chemie, Steinbach, Germany. Follicular viability was determined 24 h after tissue preparation and thawing/rapid warming. Prealiquoted calcein (Merck, Darmstadt, Germany) was dissolved with prewarmed DPBS CTS (Gibco Life technologies, NY, U.S.A.) and transferred to prealiquoted collagenase type 1A (Merck, Darmstadt, Germany) to obtain a viability working solution of 2 µmol/l calcein AM and 1 mg/ml collagenase type 1A. 2 × 2 mm biopsy punches were added to 500 µl of viability working solution and incubated for 90 min at 37.2 °C, protected from light. The solution was resuspended cautiously after 60 and 70 min to enable evenly bottoming of follicles prior fluorescence measurements at room temperature. Number of vital follicles was determined with fluorescence microscopy (Nikon, Ti2, Düsseldorf) as indicated in Fig. 6.

## Results

In all treatment groups, follicular viability was not normally distributed. As indicated in Table 1, thawing results were statistically not significant comparing thawed slow frozen and rapid warmed vitrified samples. High follicular survival rates were obtained with both cryopreservation methods, resulting in statistically non-significant differences in comparison to fresh samples, data shown in Tables 2, 3. Cryopreservation was significantly less time consuming with vitrification compared to slow freezing as indicated in Table 4. In the fresh group and in the thawed after slow freezing group, 2 different cases with absent follicular viability were observed, while in the rapid warmed after vitrification group no samples with absence of follicular viability were detected.

**Table 1:** Follicular viability after slow freezing/thawing and vitrification/rapid warming

Parameter	Thawed after slow freezing [mean]	Interval SD median	Rapid warmed after vitrification [mean]	Interval SD median	*n*	*P value
Follicular viability count [*n*]	68.6	0–454	59.8	0.5–349	30	0.459
		102.9		77.2		
		33.8		38.5		

*Wilcoxon test

**Table 2:** Follicular viability pre and post vitrification/rapid warming

Parameter	Fresh [mean]	Interval SD median	Rapid warmed after vitrification [mean]	Interval SD median	*n*	*P value
Follicular viability count [*n*]	72.8	0–386	59.8	0.5–349	30	0.900
		94.3		77.2		
		41.5		38.5		

*Wilcoxon test

**Table 3:** Follicular viability pre and post slow freezing/warming

Parameter	Fresh [mean]	Interval SD median	Thawed after slow freezing [mean]	Interval SD median	*n*	*P value
Follicular viability count [*n*]	72.8	0–386	68.6	0–454	30	0.829
		94.3		102.9		
		41.5		33.8		

*Wilcoxon test

**Table 4:** Cryopreservation time with slow freezing and vitrification

Parameter	Slow freezing method [mean]	Interval SD median	Vitrification method [mean]	Interval SD median	*n*	*P value
Cryopreservation time [min]	188.1	186–190	17	17*	30	< 0.001
		1.4		0*		
		187.5		17*		

*Mann–Whitney *U* test. *The vitrification protocol enables exact freezing time with a variation of 60 s in the final step of equilibration. This variation was not recorded and is statistically negligible

## Discussion

The aim of our study was to design and validate a high-throughput vitrification approach for human ovarian tissue based on the successful protocol of Suzuki and colleagues reporting two deliveries [26].

The technical advantage of the vitrification procedure is avoidance of ice crystal formation contrary to conventional freezing approaches [38–40]. The avoidance of ice crystal formation prevents the cellular damage, which can otherwise impair tissue integrity or even follicular survival.

Our results indicate that follicular viability rates are comparable to our standard slow freezing approach, suitable for a clinical application As a carrier system, we used cell meshes consisting of metal, linked with caps of 1.8 ml cryovials, suitable for fast loading of tissue fragments prior rapid vertical vitrification in grid embedded cryovials resulting in a high-throughput process. This is important, because in a clinical laboratory application, the amount of tissue and the numbers of tissue fragments for cryopreservation may variate substantially per patient. Processing time per each fragment is critical to minimize prolonged exposition to a high ratio of cryoprotectants in the final step of equilibration solution—resembling the vitrification process of oocytes, pronuclear (PN) stages or embryos. Additionally, the use of meshes may provide faster cooling rates compared to plastic based systems and can be sterilized by autoclavation. Our system is universally applicable for cortex stripes measuring 10 × 5 × 1 mm and for biopsy punches measuring 2 × 2 mm.

The protocol of Suzuki includes a carrier system consisting of four fine stainless needles linked with the cap of a cryogenic vial, before immersion into liquid nitrogen and insertion into cryovials after vitrification [26].

Partially, our handling approach resembles the protocol of Nikiforov, using unlinked metal meshes for carrying ovarian tissue samples with 0.28 mm wire diameter and mesh aperture of 1.31 mm for vitrification and insertion into cryovials after vitrification [41].

Our approach included metal meshes with wire diameters of 0.25 mm and opening sizes of 0.38 mm. This ensures a large contact area of the cortex tissue on a metal carrier as thin as possible, potentially influencing the thermal conductivity while the meshes were pre linked with the caps of the cryovials. This enables the use of grid embedded cryovials, pre-filled with liquid nitrogen ready for fast vertical insertion of loaded meshes into the cryovials directly during the process of vitrification. This results in a standardized, high-throughput vitrification process of ovarian cortex tissue.

One major key aspect to assess the quality of ovarian cortex tissue is follicular viability that can be evaluated with calcein, a well-described fluorescence-based live assay [42–44]. The incubation period of 24 h prior viability measurements of fresh, slow frozen/thawed and vitrified/rapid warmed tissue enables the tissue to express potential damage that could potentially occur with these procedures and may potentially not be observed when analyzed at an earlier stage. The different types of cell death can take minutes to hours [45] and might become visible later than directly after thawing/rapid warming.

Using 2 × 2 mm biopsy samples for evaluation of follicular viability provides valuable, but limited insights due to the fact that follicular density is unevenly [46–48] distributed in the human ovary potentially influencing the results. As indicated by our results, in the rapid warmed after vitrification group, no cases with absent follicular viability were observed while in the fresh and thawed after slow freezing groups 2 different samples with absence of follicular vitality were detected. To minimize these effects, a cohort of 30 patients with individual determination of follicular viability prior cryopreservation, post slow freezing and vitrification was evaluated by two experienced embryologists to mitigate researcher specific bias. In a clinical setting, preparation of at least 2 × 2 mm biopsy samples for individual quality control assessment prior and post freezing is recommended.

The design of our experimental setup is limited by the fact that the amount of tissue per patient variates substantially and our ethical vote restricts research to 10% of the amount of tissue, reflecting the limited access of researchers to ovarian tissue in a routine setting [49]. It will be interesting to investigate other tissue-specific quality markers with potential impact on implantation success like angiopoietic factors after tissue culture in a future study with a larger sample number and selected patients with larger amounts of ovarian tissue available for research purposes.

In terms of economical parameters, our results implicate that vitrification is less cost demanding regarding cryopreservation time that can have a major impact on personnel deployment planning—keeping in mind that in a busy cryobank ovarian tissue from external referrer centers scheduled for, e.g., overnight transportation [50] may occasionally arrive on the next day late in the afternoon during the week, or on saturdays, prior, e.g., a slow freezing process lasting for several hours.

Contrary to vitrification, the time consuming slow freezing cooling process must not be interrupted due to potential technical errors related with the programmable freezer unit, computer or software malfunctions and continuous nitrogen supply that could have a major impact on tissue integrity.

Additionally, slow freezing is cost intensive regarding purchasing and maintenance of equipment like controlled freezing systems with an attached nitrogen supply tank, personal computer, freezing software and requires in contrast to vitrification security of electricity during the cooling procedure.

In summary, our results indicate that rapid vertical vitrification of ovarian tissue may be equivalent to slow freezing in terms of follicular viability while offering a cost-efficient alternative to conventional slow freezing procedures. We follow the argumentation of Suzuki [26], Keros [51], Sugishita [52], Silver [53] and Nikiforov [41] that vitrification of ovarian tissue is a promising alternate approach to conventional slow freezing systems for ovarian tissue.
